# Delayed Tibial Shaft Fracture Healing Associated with Smoking: A Systematic Review and Meta-Analysis of Observational Studies Conducted Worldwide

**DOI:** 10.3390/ijerph181910228

**Published:** 2021-09-28

**Authors:** Akanksha Mahajan, Narinder Kumar, Bhawna Gupta

**Affiliations:** 1 BMed Sci/MD, Monash University, Melbourne, VIC 3168, Australia; amah0039@student.monash.edu; 2 Department of Orthopaedics, Medanta Hospital, Lucknow 226030, India; 3 Department of Public Health, Torrens University, Melbourne, VIC 3000, Australia; bhawna.gupta@torrens.edu.au

**Keywords:** smoking, tibia, fracture, tibial shaft fracture, fracture healing, cigarette

## Abstract

Tibial fractures represent a great burden of disease globally, being the most common long-bone fracture; smoking is a known risk factor for delayed skeletal healing and post-fracture complications. This systematic review and meta-analysis aims to analyse the effect of smoking on healing of tibial shaft fractures. PubMed, CINAHL, EMBASE, and Cochrane Library databases were searched from inception to March 2021, with no limitation on language, to find relevant research. All observational studies that assessed the association between cigarette smoking and tibial shaft fracture healing in adults (≥18 years) were included. The quality of studies was evaluated using the Newcastle Ottawa Quality Assessment Scale. A random effects model was used to conduct meta-analysis. Tobacco smoking was associated with an increased rate of non-union and delayed union as well as an increase in time to union in fractures of the tibial shaft. Among the 12 included studies, eight reported an increased rate of non-union, three reported delayed union, and five reported an increase in time to union. However, the results were statistically significant in only three studies for non-union, one for delayed union, and two studies for increased time to union. This review confirms the detrimental impact of smoking on tibial shaft fracture healing and highlights the importance of patient education regarding smoking cessation.

## 1. Introduction

Tibial fractures are the most common long-bone fracture [1] and represent a significantly large proportion of skeletal injuries. These fractures are most often caused by road traffic accidents and sports activities, with males being more likely to suffer high energy trauma from sports activity and women being more likely to sustain low energy injuries [2,3,4]. There is a huge burden of disease as well as economic burden associated with tibial shaft fractures due to direct medical costs as well as lost productivity, including workplace absences and short-term disability, particularly in younger patients [5].

Fractures heal through a complex mechanism affected by multiple biological, mechanical, local and systemic factors. There are multiple modifiable and non-modifiable risk factors which are deleterious to fracture healing, including high energy trauma, open fractures, a high degree of initial fracture displacement, inadequate stability, infection, presence of a post-surgical fracture gap, age, smoking, diabetes, obesity, and NSAID use [6,7]. Moreover, the larger subcutaneous border of the tibia in comparison to other longer bones leads it to have a relatively poor blood supply, increasing the risk of delayed and non-union healing in tibial fractures [8]. In this review, non-union was defined as no sign of union at 9 months or later, while delayed union was defined as incomplete union at 3 months, which was completed by 6 months after initial injury [9]. An increase in time to union was evaluated in weeks when comparing union time in smokers and non-smokers.

Despite the morbidity and mortality associated with smoking being well known, it remains a common addiction all over the world. Smoking is one of the most common and important modifiable factors associated with delayed skeletal healing and post-fracture complications [10]. According to the “diamond concept” introduced by Giannoudis et al., successful fracture healing is dependent on three factors in the biological environment at the fracture site (availability of osteoinductive mediators, osteogenic cells, and an osteoconductive matrix) as well as a fourth factor called mechanical stability [11]. Smoking is likely to have an influence on all the biological factors within this model and thereby impair fracture healing.

Many observational studies, including systematic analyses, have explored the effect of smoking on tibial fracture healing [9,12]. This is the first systematic review and meta-analysis aimed at exploring and assessing the available evidence on the relationship of smoking as a risk factor for delayed union, non-union, and risk of infection as well as the time taken for union in fractures of shaft of tibia, comparing smokers with non-smokers. The protocol for this review was registered in PROSPERO (registration number CRD42021239556). This review will provide valuable information and an evidence base for future patient education regarding smoking as a risk factor for impaired healing of tibial shaft fractures.

## 2. Materials and Methods

### 2.1. Search Strategy

The PubMed, CINAHL, EMBASE, and Cochrane Library databases were systematically searched for observational studies (case-control, cohort, cross-sectional) that assessed the association between cigarette smoking and tibial shaft fracture healing from inception to March 2021, with no limitation on language.

We followed Preferred Reporting Items for Systematic Review and Meta-Analysis (PRISMA) strategy [13]. The Critical Appraisal Skills Programme checklist was used to assess the association between smoking and tibial shaft fracture healing.

Meta-analysis of Observational Studies in Epidemiology (MOOSE) guidelines were followed to conduct the meta-analysis [14]. The following search keywords were used with Boolean operators to combine searches: (smok*) OR (cigarette) AND (tibia*) AND (fracture). A health librarian was consulted for a review on our search strategy. Subsequently, a manual search was performed by checking the reference lists of key studies and review articles, as well as the use of “related citations” function in PubMed, to identify additional studies. Active surveillance of databases using alerts with search strategies was also conducted while drafting the manuscript. Efforts were made to contact the corresponding authors of articles which were not freely available or were in other languages and could not be translated to English. In order to remove duplicate articles from different databases, EndNote X9 software (Clarivate, Philadelphia, PA, USA) was utilised [15].

### 2.2. Study Selection

An overall literature search was performed, and relevant studies were screened independently by two reviewers (AM and NK). Initially, all the titles and abstracts which were identified based on the keywords were screened. Second, full texts of articles which were selected from the first phase were reviewed. Finally, those articles which had contents suitable for data extraction were included in the systematic review. Corresponding authors were contacted by mail for the papers that were not freely available to us through the databases. Grey literature was also explored for emerging findings.

### 2.3. Exclusion Criteria

Exclusion criteria were as follows: papers published in a language other than English for which translation was unavailable; animal model experiments; studies with patients < 18 years of age; insufficient information; review articles and case reports; studies with a follow-up period of less than 12 months.

### 2.4. Data Extraction

Relevant data were extracted independently by two reviewers (AM and NK). The following information was entered into a pre-designed form: paper citation, study time frame, study design, region, sample population, follow-up period, patient demographics (including age, gender), fracture type, operative information, the number of smokers and number of non-smokers, definition of tibial shaft fracture, definition of smoking, and adjusted covariates in the regression model. The smokers were defined on the basis of their self-reported history, and data was also extracted from patients’ hospital records. The definition of smokers in the included studies ranged from: any history of smoking, ≥5 to ≥10 cigarettes per day and ≥100 cigarettes over lifetime. Disagreements between the two reviewers were resolved by a third reviewer (BG) via discussion and consensus. In studies for which odds ratios were not given but adequate information was provided [16,17,18], two reviewers (BG and AK) calculated the odds ratios for inclusion in meta-analysis using the software MedCalc, (MedCalc Software Ltd., Ostend, Belgium) available at https://www.medcalc.org/calc/odds_ratio.php (accessed on 21 July 2021).

### 2.5. Quality Assessment

The methodological quality of studies was evaluated using the Newcastle Ottawa Quality Assessment Scale. This tool includes 8 items, categorised into the following 3 categories: Selection, Comparability, and Outcome (for cohort studies) or Exposure (for case-control studies) [19]. Each of these is subdivided into 1, 3, and 4 items, respectively, which act as the basis for assignment of stars. The total stars awarded on this scale can range from zero to nine stars, with the highest quality studies being awarded a maximum of one star per item, with the exception of the item related to comparability, which allows two stars to be awarded [19].

### 2.6. Data Analysis and Summary Estimates

Comprehensive meta-analysis software (Biostat Inc. Englewood, NJ, USA) was used for all analyses [20]. A random effects model was used to conduct meta-analysis and to produce forest plots. The forest plots in this study were used to demonstrate the effect of each study and the summary effect size. The reported estimates of 95% confidence intervals (CI) were used to calculate the standard errors of the logarithm and the effect size estimates. For each random effects meta-analysis, heterogeneity was assessed by using Cochran’s Q statistic (measure of weighted square deviations), with N − 1 degrees of freedom (where N is the number of studies), results of statistical test based on Q statistic, between studies variance (T2), and ratio of the true heterogeneity to total observed variation (I2). For the studies that reported both adjusted and crude estimated ORs, the adjusted effect estimates were used in this study for meta-analysis.

Funnel plots were created to graphically present the publication bias. The distribution of study risk estimates across the funnel plot was examined visually and Egger’s test for small study effects was performed to assess the degree of asymmetry when *p* < 0.05.

## 3. Results

**Figure 1:**
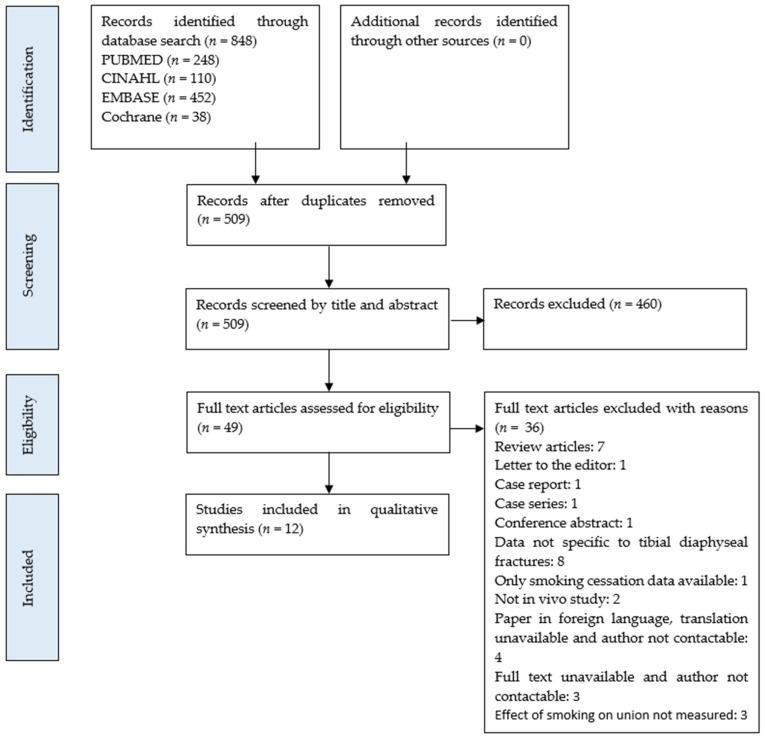
PRISMA Flowchart.

The PRISMA flowchart (Figure 1) illustrates that a total of 848 publications were yielded from the database searches, hand search of reference lists, and grey literature. Two hundred and forty-eight hits were found from PubMed with an addition of 110 from CINAHL, 452 from EMBASE, and 38 from Cochrane. After removing duplicate records, a total of 509 articles remained. After reading the titles and abstracts, 463 studies were removed, with 46 remaining that were assessed for full eligibility. Upon reading the full text of each article and removing those that did not fit the inclusion criteria, a total of 12 studies remained. Studies were excluded due to unrelated outcomes, non-not being in vivo experiments, or being review articles, letters to editors or comments, conference abstracts, case reports/series, as well as the translation unavailability of non-English articles, with further details provided in the PRISMA flowchart. The summary and characteristics of these articles are included in Table 1 and Table 2.

### 3.1. Characteristics of Included Studies

There are twelve cohort studies included in this systematic review, which were published between 1999 and 2020. The majority of these studies included populations from Europe [16,17,18,21,22,23,24]. Three studies were conducted in the United States of America [25,26,27]. The sample size varied from 32 [21] to 940 participants [26].

Although smoking was the main determinant studied in all the included studies, the study designs were not uniform or clearly defined. Six studies were prospective [17,21,25,26,27,28], and six were retrospective [16,18,22,23,24,29]. There was no overlap of patients/centres between any of the studies.

Among these studies, one was conducted on delayed union [25], while three measured non-union [16,18,26], and eight studies reported both. This systematic review included 1158 smokers and 1894 non-smokers, with a total of 3052 participants. Castillo et al.’s work [25] was the only study of those included in this review which differentiated between current and previous smoking when reporting smoking status. There was one study which defined smoking as ≥5 cigarettes smoked per day [27]. Other studies defined it as smoking ≥10 cigarettes per day [16] and ≥100 cigarettes over a lifetime [25]. Five studies did not clearly define “smokers” and considered any history of smoking [21,23,26,28,29], while four studies classified participants as current/prior/non-smokers [17,18,22,24]. None of the studies evaluated nicotine levels in participants or reported on the use of smokeless tobacco. The overall age group of study participants ranged from 13 years to 90 years [17,21,22,23,24,27,28,29]. Most of these studies included both males (total of 2442) and females (total of 578), save one in which gender was unspecified [21]. Only six studies adjusted for potential confounding factors with the use of a multivariate/binary logistic regression [17,22,23,24,26,28].

For the meta-analysis on the effect of smoking on time to union, eight studies were included [16,17,18,22,23,24,26,28], with a total sample size of 2301, including 806 smokers and 1495 non-smokers.

### 3.2. Quality Assessment

The included studies all had a score of at least 5 on the Newcastle–Ottawa Scale for Quality Assessment, and the results are displayed in Table 3. The minimum and maximum scores were 5 and 9, respectively. Five studies attained the maximum score of 9 [24,25,26,27,28]. The domain of comparability on the basis of design or analysis was associated with the lowest scores, which indicates the possibility of bias in studies in which there was no adjustment for confounding variables.

### 3.3. Effect of Smoking on Tibial Shaft Fracture Non-Union and Healing Times

Time to union in both smokers and non-smokers was measured in five of the included studies [16,17,21,22,25]. The mean time to union in smokers was 28.61 weeks, whereas in non-smokers it was 22.03 weeks.

### 3.4. Meta-Analysis for Non-Union

**Figure 2:**
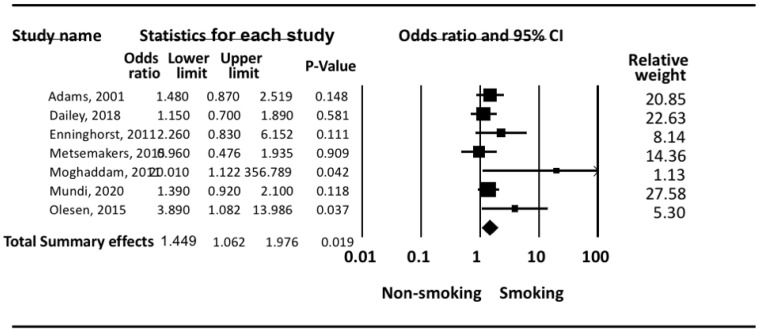
Meta Analysis for non-union.

In Figure 2: the odds of tibial shaft fracture non-union as compared to fractures which achieved union were greater among smokers as compared to non-smokers. Smoking increased the risk of non-union significantly (*p* = 0.019). Under the random effects model, the overall pooled estimate risk for non-union was (OR: 1.45; 95% CI: 1.06–1.98 *p* < 0.05).

The highest risk estimates observed were (OR: 20.01; 95% CI: 1.12–356.79, *p* < 0.05) in a study conducted in Germany from 2002 to 2005 on 46 smokers and 39 non-smokers [17]. However, the wide 95% CI indicates that the small sample size is a limitation of this study.

### 3.5. Meta-Analysis for Delayed Union

**Figure 3:**
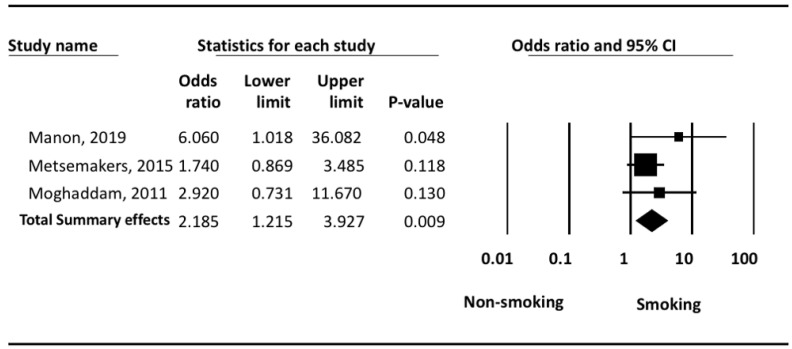
Meta Analysis of delayed union.

In Figure 3: the odds of delayed union of tibial shaft fracture as compared to timely union were greater among smokers as compared to non-smokers. Smoking increased the risk of delayed union significantly (*p* = 0.009). Under the random effects model, the overall pooled estimate risk for delayed union was (OR: 2.19; 95% CI: 1.21–3.93, *p* < 0.05).

The highest risk estimates observed were (OR: 6.06; 95% CI: 1.02–36.08, *p* = 0.048) in a study conducted in Belgium from 2005 to 2015 on 40 smokers and 131 non-smokers [23].

### 3.6. Publication Bias and Meta-Regression

**Figure 4:**
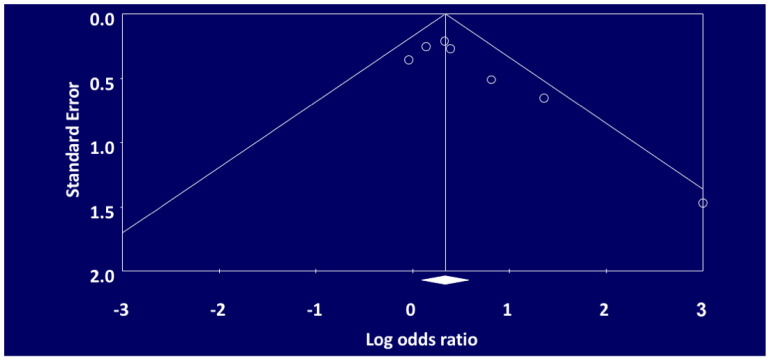
Funnel Plot of Standard Error by Logs Ratio for Non-Union.

**Figure 5:**
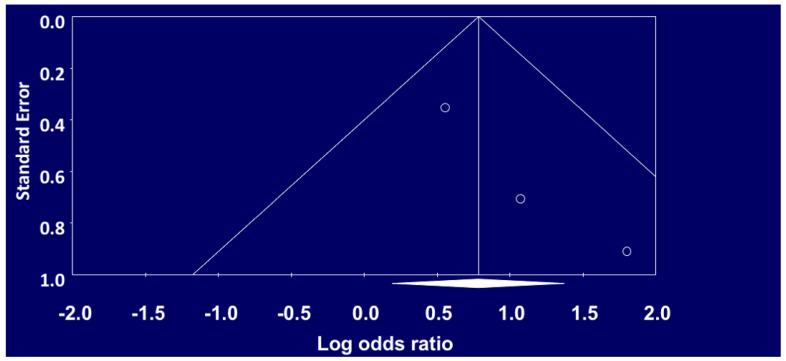
Funnel Plot of Standard Error by Logs Ratio for Delayed Union.

The funnel plot constructed for non-union of tibial fractures in smokers as compared to non-smokers is symmetrical (Figure 4, indicating that publication bias was not present. The asymmetrical funnel plot (Figure 5) by visual inspection for delayed union indicates that publication bias was present in our meta-analysis for this outcome.

## 4. Discussion

### 4.1. Summary and Significance of Main Results

This is the first systematic review aimed at exploring the relationship between smoking and time to union in fractures of the shaft of the tibia. The results of this review would be clinically relevant to the routine practice of orthopaedic surgeons as well as general practitioners globally, considering that smoking is a ubiquitous health hazard.

It is evident that tobacco smoking was associated with an increased rate of non-union and delayed union as well as an increase in time to union in fractures of the shaft of the tibia. Among the twelve included studies, eight studies reported an increased rate of non-union [16,17,18,22,25,26,28,29], three studies reported delayed union [17,23,24] and five studies reported an increase in time to union in fractures of the shaft of the tibia [16,17,21,25,27]. However, the results were statistically significant in only three studies for non-union [17,18,29], one study for delayed union [23], and in two studies for increased time to union [25,27]. It is likely that there is a temporal relationship between smoking and non-union, delayed union, and/or increased time to union of tibial shaft fractures.

Similar results were reported in a systematic review by Tian et al. which reported that the prevalence of tibial fracture non-union was significantly higher in smokers as compared to non-smokers (*p* = 0.111) [9]. Pearson et al. also reported that smoking is linked to an increased risk of delayed union and/or non-union, finding that when considered collectively, smokers had 2.2 times the risk of experiencing delayed union and/or non-union, and that smoking was associated with an increase in time to union of 27.7 days [12].

The toxic by-products of smoking such as nicotine and carbon monoxide significantly impair bone healing through interference with neovascularization and collagen synthesis as well as osteoblast production and differentiation [30]. Nicotine decreases tissue perfusion due to increased platelet aggregation and decreased microvascular prostacyclin levels as well as its inhibitory effects on the function of fibroblasts, red blood cells and macrophages [31]. Additionally, carbon monoxide has a high binding affinity for haemoglobin, allowing it to lower tissue oxygenation by displacing oxygen from haemoglobin [31]. This explains the biological plausibility of delayed tibial shaft fracture healing due to smoking habits.

### 4.2. Study Participants

All the studies were conducted in high-income countries: seven in Europe [16,17,18,21,22,23,24], three in the United States of America [25,26,27] and one each in Singapore [29] and Australia [28]. This creates an inadvertent bias as the quality and accessibility of clinical care available to the patients in these countries are significantly better than in lower income countries.

### 4.3. Limitations of This Review

Most of the studies included did not report quantitative data on smoking including the frequency, duration, or intensity of an individual’s smoking habits, with most studies classifying the patients as either “smokers” or “non-smokers”. As a result, the presence of a dose–response relationship between smoking and delayed union/non-union cannot be evaluated. It is likely that the results of these studies are influenced by other contributing factors including confounding and bias. Measuring nicotine levels can enable quantification of smoking, but none of the studies evaluated nicotine levels in participants. Furthermore, not a single study reported on the use of smokeless tobacco. This is a gap in the research which future studies should be directed towards addressing.

Moreover, six of the included studies were retrospective cohort studies, which introduces risk of bias and confounding, limiting the quality of the data extracted from them. In addition, only six of the studies adjusted for comorbidities such as age, diabetes, gender, mechanism of injury, nature of injury (open versus closed), etc. The remaining six studies therefore have a high risk of confounding, which may have decreased the quality of the data. Quality analysis of the included studies, as well as meta-analysis, has also shown the presence of significant publication bias.

Although patient co-morbidities have known effects on fracture healing, we were not able to analyse the effect of patient co-morbidities as these were either not recorded or controlled/adjusted for in the majority of the evaluated studies.

## 5. Conclusions

This review analyses and further supports the body of evidence highlighting the detrimental effect of smoking on the healing of tibial shaft fractures. It is recommended that the detailed history of smoking should be a part of detailed clinical history, including frequency and duration of use as well as the nature and quantity of tobacco product being consumed. In view of the risk of an increase in time to union/delayed union posed by smoking in patients with fractures of the shaft of the tibia, it is important to discuss the same with the patient at the time of planning their treatment. The beneficial effect of cessation of smoking on various surgical procedures is well established, and this can be a part of the patient counselling process [32,33].

It will be valuable to conduct prospective studies in future to measure the dose–response relationship of effects of smoking on tibial fracture healing. Furthermore, the effect of smokeless tobacco products needs further study considering their systemic delivery of nicotine.

Considering the global burden of tibial fracture care, this analysis provides an opportunity for general practitioners as well as orthopaedic surgeons to encourage their patients to quit smoking, thus ensuring earlier healing as well as a decrease in the healthcare and economic resources required to manage delayed/non-union.

## Figures and Tables

**Table 1:** Demographic characteristics and health outcome of study population.

Reference	Region	Time Frame of Study	Design of Study	Follow Up	Gender	Age in Years (Mean/ST Dev or Range)	Health Outcome Definition
Adams, Keating and Court-Brown, 2001 [16]	Netherlands	1983–1995	Retrospective cohort	Mean 21.6 months	M (112) F (61)	(Range, 13–90); 38.7 smokers, 39.2 non-smokers	Non-union following open tibial fractures
Alemdaroglu et al., 2009 [21]	Turkey	2002–2007	Prospective cohort	Monthly, for at least 6 months	Unspecified	Mean 45.3 (range 19–75)	Time to fracture healing, delayed union, non-union
Castillo et al., 2005 [25]	United States of America	1994–1997	Prospective cohort	3 monthly for 24 months	M (246) F (209)	Mean 33.4 (range 16–69)	Time to fracture healing
Dailey et al., 2018 [22]	Scotland	1985–2007	Retrospective cohort	Not mentioned	M (739) F (264)	Mean males = 31.3, mean females = 45.1	Time to healing and non-union rates after reamed intramedullary nailing
Enninghorst et al., 2011 [28]	Australia	2007–2009	Prospective cohort	12 months	M (66) F (23)	41 ± 17	Assessment of both non-union risk and time to union
Manon-et al., 2019 [23]	Belgium	2005–2015	Retrospective cohort	9 months	M (105) F (66)	Mean 45.6, range: 14–95	Time to fracture healing and delayed union rates
Metsemakers et al., 2015 [24]	Belgium	2000–2012	Retrospective cohort	Minimum 18 months, until evidence of union	M (338) F (142)	Mean 39.2, range 17–90	Compromised fracture healing: Delayed union; non-union; requirement of secondary procedure
Moghaddam et al., 2011 [17]	Germany	2002–2005	Prospective cohort	Mean 40 months	M (61) F (24)	Mean 46, range: 18–84 at the time of injury	Time to fracture healing, delayed union, non-union
Mundi et al., 2020 [26]	United States, Canada, and The Netherlands	2000–2005	Prospective cohort	12 months	M (709) F (231)	40.9 ± 15.6	Non-union rates
Olesen et al., 2015 [18]	Denmark	2002–2013	Retrospective cohort	12 months	M (32) F (13)	42 ± 18, range 16–71	Non-union rates
Schmitz et al., 1999 [27]	USA	1990–1993	Prospective cohort	12 months	M (73) F (30)	Smoker: 35.6 ± 1.7, Non-smoker: 35.8 ± SD 1.9	Time to clinical union, non-union, time to radiographic healing
Singh et al., 2018 [29]	Singapore	2000–2013	Retrospective cohort	Minimum 6 months, until union	M (111) F (8)	38.2, range 18–70	Time to fracture union and non-union

**Table 2:** Demographic characteristics and exposure to smoking of study population.

Reference	Exposure to Smoking	Non-Exposed to Smoking	Smoking Habit by Gender	Definition of Smoking	Adjusted Odds Ratio and 95% CI and *p* Value	Healing Time Smokers in Weeks, Mean ± SD	Healing Time Non-Smokers, Mean ± SD in Weeks
Adams, Keating and Court-Brown, 2001 [16]	140 smokers	133	12 males, 28 females smokers; 100 males, 33 females non-smokers	10 or more cigarettes per day, not intermittently	OR 1.48 (95% CI 0.87 to 2.51) non-union, *p* = 0.14	32.3	27.8
Alemdaroglu et al., 2009 [21]	13 smokers	19	Not mentioned	Not defined	*p* = 0.158	27.54 ± 11.609	21.37 ± 5.079
Castillo et al., 2005 [25]	82 previous smoker, 105 current smoker	81	73% males, 27% females	Never smoked, previous smoker (100 or more cigarettes over the course of his or her lifetime), current smoker	Current smokers versus non-smokers (*p* = 0.01), whereas previous smokers versus non-smokers (*p* = 0.04)	47.8 previous smoker, 42.9 current smoker	40.1
Dailey et al., 2018 [22]	244 smokers	261	739 males, 264 females	Patients with records for smoking	Smoker OR: 1.15; 95% CI: 0.70–1.89, *p* = 0.572 for non-union rate, *p* = 0.006 for time to union	18	18
Enninghorst et al., 2011 [28]	31 smokers	90	74% (66) male, 26% females	Not mentioned	Non-union: OR: 2.26; 95% CI: 0.83–6.15	Not mentioned	Not mentioned
Manon-et al., 2019 [23]	40 smokers	131	105 males and 66 females	Not defined	Delayed union: OR: 6.06; 95% CI: 1.02–36.16, *p* = 0.048	Not mentioned	Not mentioned
Metsemakers et al., 2015 [24]	146 smokers	334	338 male patients (70.4%) and 142 female patients (29.6%)	Active smokers at time of the initial procedure	Delayed union: OR: 1.74; 95% CI: 0.87–3.49, *p* = 0.120; non-union: OR: 0.96; CI 0.48–1.95, *p* = 0.915	Not mentioned	Not mentioned
Moghaddam et al., 2011 [17]	46 smokers	39	61 men (72%) and 24 women (28%)	Self-reported smoking status	Delayed union OR: 2.92; 95% CI: 0.73 to 11.65; Non-union OR: 20.01, 95% CI: 1.125 to 356.08 *p* = 0.0007	17.4	11.9
Mundi et al., 2020 [26]	299 smokers	640	709 males, 231 females	Not defined	Non-union: OR: 1.39; CI: 0.92–2.10, *p* = 0.113	Not mentioned	Not measured
Olesen et al., 2015 [18]	15 smokers	30	13 women and 32 men	Data from patient records regarding tobacco use	Non-union: OR: 3.89, 95% CI: 1.08–13.96, *p* ≤ 0.058	Not mentioned	Not measured
Schmitz et al., 1999 [27]	76 smokers	59	73 (31 smoker, 13 non-smoker) males, 30 (13 smoker, 17 non-smoker) females	Smoke more than 5 cigarettes per day at the time of fracture	Not mentioned	Not mentioned	Not mentioned
Singh et al., 2018 [29]	26 smokers	77	111 males (93.2%) and eight females	Not defined	Not mentioned	Revision (due to non-union)/delayed union in smokers versus smokers *p* = 0.0381	

**Table 3:** Quality Assessment of the Studies by the Newcastle-Ottawa Scale^18^).

Reference	Overall Quality Assessment-Max 9	Selection				Comparability	Outcome		
		Representativeness	Selection of Non-Exposed Cohort	Ascertainment of Exposure	Outcome of Interest Was Not Present at Start of Study		Assessment	Duration of Follow Up	Adequacy of Follow Up (>80%)
Adams, Keating and Court-Brown, 2001 [16]	7	1	1	1	1	0	1	1	1
Alemdaroglu et al., 2009 [21]	5	1	1	0	1	0	1	0	1
Castillo et al., 2005 [25]	9	1	1	1	1	2	1	1	1
Dailey et al., 2018 [22]	8	1	1	1	1	2	1	0	1
Enninghorst et al., 2011 [28]	9	1	1	1	1	2	1	1	1
Manon-et al., 2019 [23]	8	1	1	1	1	2	1	1	Unspecified- retrospective study
Metsemakers et al., 2015 [24]	9	1	1	1	1	2	1	1	1
Moghaddam et al., 2011 [17]	8	1	1	0	1	2	1	1	1
Mundi et al., 2020 [26]	9	1	1	1	1	2	1	1	1
Olesen et al., 2015 [18]	6	1	1	1	1	0	1	1	Unspecified- retrospective study
Schmitz et al., 1999 [27]	9	1	1	1	1	2	1	1	1
Singh et al., 2018 [29]	7	1	1	1	1	0	1	1	1

## Data Availability

All the data available has been presented in this manuscript.

## References

[1] Kojima K.E., Ferreira R.V. (2015). Tibial Shaft Fractures. Rev. Bras. Ortop..

[2] Court-Brown C.M., Caesar B. (2006). Epidemiology of adult fractures: A review. Injury.

[3] Larsen P., Elsoe R., Hansen S.H., Graven-Nielsen T., Laessoe U., Rasmussen S. (2015). Incidence and epidemiology of tibial shaft fractures. Injury.

[4] Madadi F., Farahmandi M.V., Eajazi A., Besheli L.D., Madadi F., Lari M.N. (2010). Epidemiology of adult tibial shaft fractures: A 7-year study in a major referral orthopedic center in Iran. Med. Sci. Monit..

[5] Bonafede M., Espindle D., Bower A.G. (2013). The direct and indirect costs of long bone fractures in a working age US population. J. Med. Econ..

[6] Nicholson J.A., Makaram N., Simpson A., Keating J.F. (2020). Fracture nonunion in long bones: A literature review of risk factors and surgical management. Injury.

[7] Santolini E., West R., Giannoudis P.V. (2015). Risk factors for long bone fracture non-union: A stratification approach based on the level of the existing scientific evidence. Injury.

[8] McMillan T.E., Johnstone A.J. (2017). Technical considerations to avoid delayed and non-union. Injury.

[9] Tian R., Zheng F., Zhao W., Zhang Y., Yuan J., Zhang B., Li L. (2020). Prevalence and influencing factors of nonunion in patients with tibial fracture: Systematic review and meta-analysis. J. Orthop. Surg. Res..

[10] Scolaro J.A., Schenker M.L., Yannascoli S., Baldwin K., Mehta S., Ahn J. (2014). Cigarette Smoking Increases Complications Following Fracture: A Systematic Review. JBJS.

[11] Andrzejowski P., Giannoudis P.V. (2019). The ‘diamond concept’ for long bone non-union management. J. Orthop. Traumatol..

[12] Pearson R.G., Clement R.G.E., Edwards K.L., Scammell B.E. (2016). Do smokers have greater risk of delayed and non-union after fracture, osteotomy and arthrodesis? A systematic review with meta-analysis. BMJ Open.

[13] Page M.J., McKenzie J.E., Bossuyt P.M., Boutron I., Hoffmann T.C., Mulrow C.D., Shamseer L., Tetzlaff J.M., Akl E.A., Brennan S.E. (2021). The PRISMA 2020 statement: An updated guideline for reporting systematic reviews. BMJ.

[14] Brooke B.S., Schwartz T.A., Pawlik T.M. (2021). MOOSE Reporting Guidelines for Meta-analyses of Observational Studies. JAMA Surg..

[15] Bramer W.M. (2018). Reference checking for systematic reviews using Endnote. J. Med. Libr. Assoc. JMLA.

[16] Adams C.I., Keating J.F., Court-Brown C.M. (2001). Cigarette smoking and open tibial fractures. Injury.

[17] Moghaddam-Alvandi A., Zimmermann G., Hammer K., Bruckner T., Grützner P.A., von Recum J. (2013). Cigarette smoking influences the clinical and occupational outcome of patients with tibial shaft fractures. Injury.

[18] Olesen U.K., Juul R., Bonde C.T., Moser C., McNally M., Jensen L.T., Elberg J.J., Eckardt H. (2015). A review of forty five open tibial fractures covered with free flaps. Analysis of complications, microbiology and prognostic factors. Int. Orthop..

[19] Stang A. (2010). Critical evaluation of the Newcastle-Ottawa scale for the assessment of the quality of nonrandomized studies in meta-analyses. Eur. J. Epidemiol..

[20] Borenstein M., Rothstein H. (1999). Comprehensive Meta-Analysis.

[21] Alemdaroğlu K.B., Tiftikçi U., Iltar S., Aydoğan N.H., Kara T., Atlihan D., Ateşalp A.S. (2009). Factors affecting the fracture healing in treatment of tibial shaft fractures with circular external fixator. Injury.

[22] Dailey H.L., Wu K.A., Wu P.-S., McQueen M.M., Court-Brown C.M. (2018). Tibial Fracture Nonunion and Time to Healing After Reamed Intramedullary Nailing: Risk Factors Based on a Single-Center Review of 1003 Patients. J. Orthop. Trauma.

[23] Manon J., Detrembleur C., Van de Veyver S., Tribak K., Cornu O., Putineanu D. (2019). Predictors of mechanical complications after intramedullary nailing of tibial fractures. Orthop. Traumatol. Surg. Res..

[24] Metsemakers W.J., Handojo K., Reynders P., Sermon A., Vanderschot P., Nijs S. (2015). Individual risk factors for deep infection and compromised fracture healing after intramedullary nailing of tibial shaft fractures: A single centre experience of 480 patients. Injury.

[25] Castillo R.C., Bosse M.J., MacKenzie E.J., Patterson B.M., Castillo R.C., Bosse M.J., MacKenzie E.J., Patterson B.M. (2005). Impact of smoking on fracture healing and risk of complications in limb-threatening open tibia fractures. J. Orthop. Trauma.

[26] Mundi R., Axelrod D., Heels-Ansdell D., Chaudhry H., Ayeni O.R., Petrisor B., Busse J.W., Thabane L., Bhandari M. (2020). Nonunion in Patients with Tibial Shaft Fractures: Is Early Physical Status Associated with Fracture Healing?. Cureus.

[27] Schmitz M.A., Finnegan M., Natarajan R., Champine J. (1999). Effect of Smoking on Tibial Shaft Fracture Healing. Clin. Orthop. Relat. Res..

[28] Enninghorst N., McDougall D., Hunt J.J., Balogh Z.J. (2011). Open tibia fractures: Timely debridement leaves injury severity as the only determinant of poor outcome. J. Trauma.

[29] Singh A., Hao J.T., Wei D.T., Liang C.W., Murphy D., Thambiah J., Han C.Y. (2018). Gustilo IIIB Open Tibial Fractures: An analysis of Infection and Nonunion Rates. Indian J. Orthop..

[30] Walker L.M., Preston M.R., Magnay J.L., Thomas P.B., El Haj A.J. (2001). Nicotinic regulation of c-fos and osteopontin expression in human-derived osteoblast-like cells and human trabecular bone organ culture. Bone.

[31] Sloan A., Hussain I., Maqsood M., Eremin O., El-Sheemy M. (2010). The effects of smoking on fracture healing. Surgeon.

[32] Sørensen L.T. (2012). Wound Healing and Infection in Surgery: The Pathophysiological Impact of Smoking, Smoking Cessation, and Nicotine Replacement Therapy: A Systematic Review. Ann. Surg..

[33] Wong J., Lam D.P., Abrishami A., Chan M.T., Chung F. (2012). Short-term preoperative smoking cessation and postoperative complications: A systematic review and meta-analysis. Can. J. Anesth..

